# Supplementary material for: MethNet: a robust approach to identify regulatory hubs and their distal targets from cancer data
Source: Nat Commun. 2024 Jul 17;15:6027. doi: 10.1038/s41467-024-50380-3 (PMC11258126; doi:10.1038/s41467-024-50380-3)
Supplement: Supplementary file 1 — Supplementary Information [file 41467_2024_50380_MOESM1_ESM.pdf]

Supplementary Figure S1

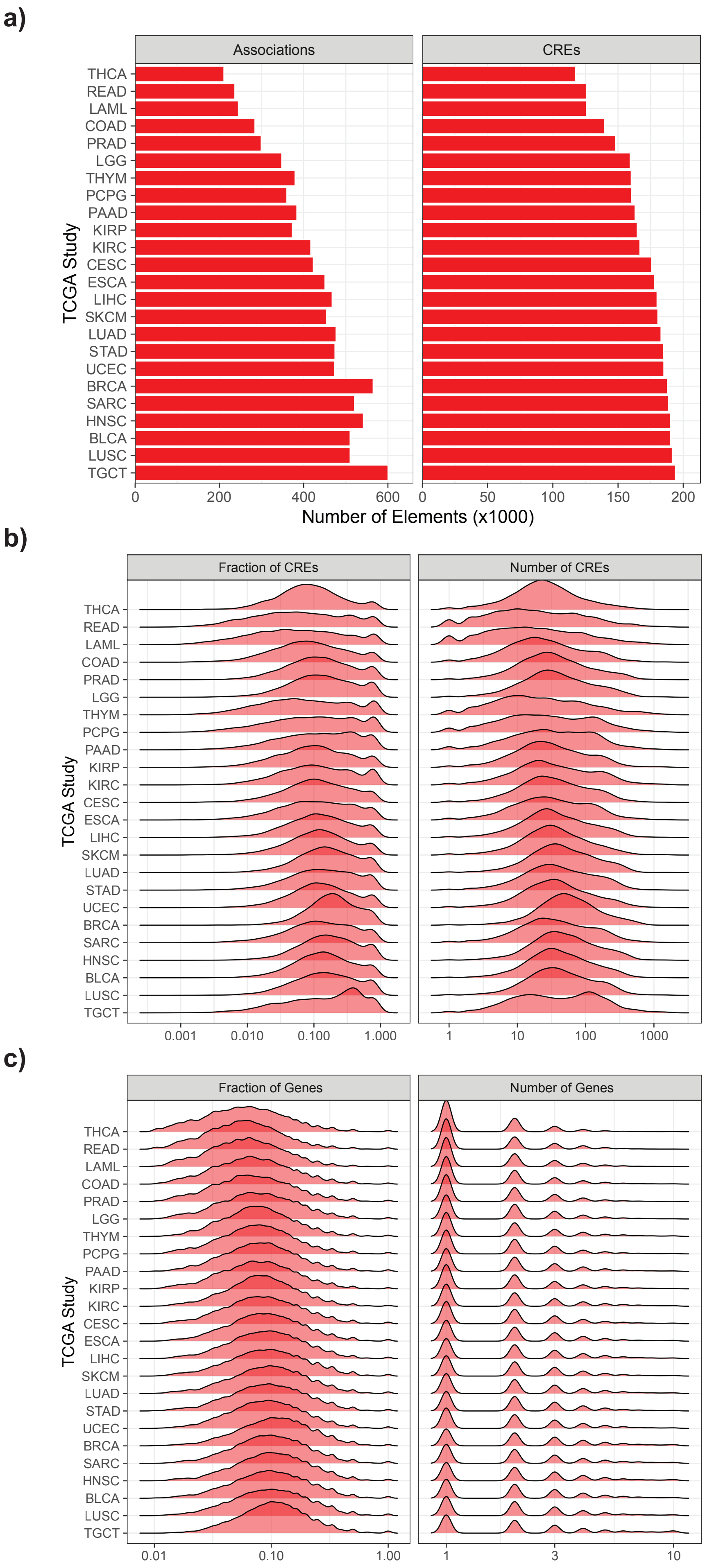

Figure S1: **Basic statistics of MethNet cancer specific regulatory networks**. **a**) Number of recovered associations and CREs per TCGA study. **b**) Distribution of fraction of the potential and absolute number of CRE (associations) per gene across TCGA studies. **c**) Distribution of fraction of the potential and absolute number of genes (associations) per CRE across TCGA studies.

Supplementary Figure S2

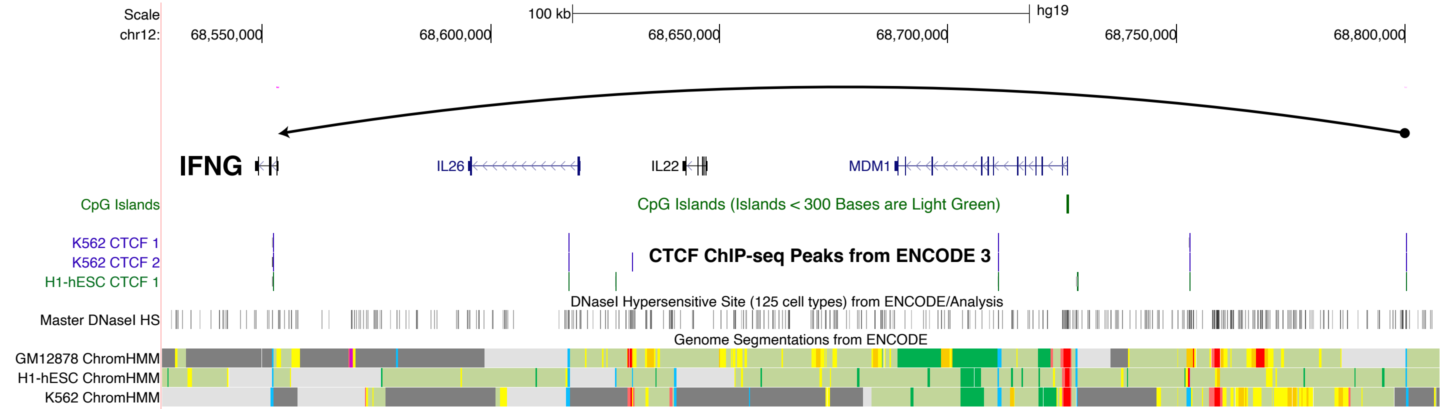

Figure S2: **An example of a repressive MethNet association**. *IFNγ* expression is regulated by a CTCF binding site located 250 kb upstream of its promoter. UCSC Genome Browser session highlighting the MethNet association on top track.

Supplementary Figure S3

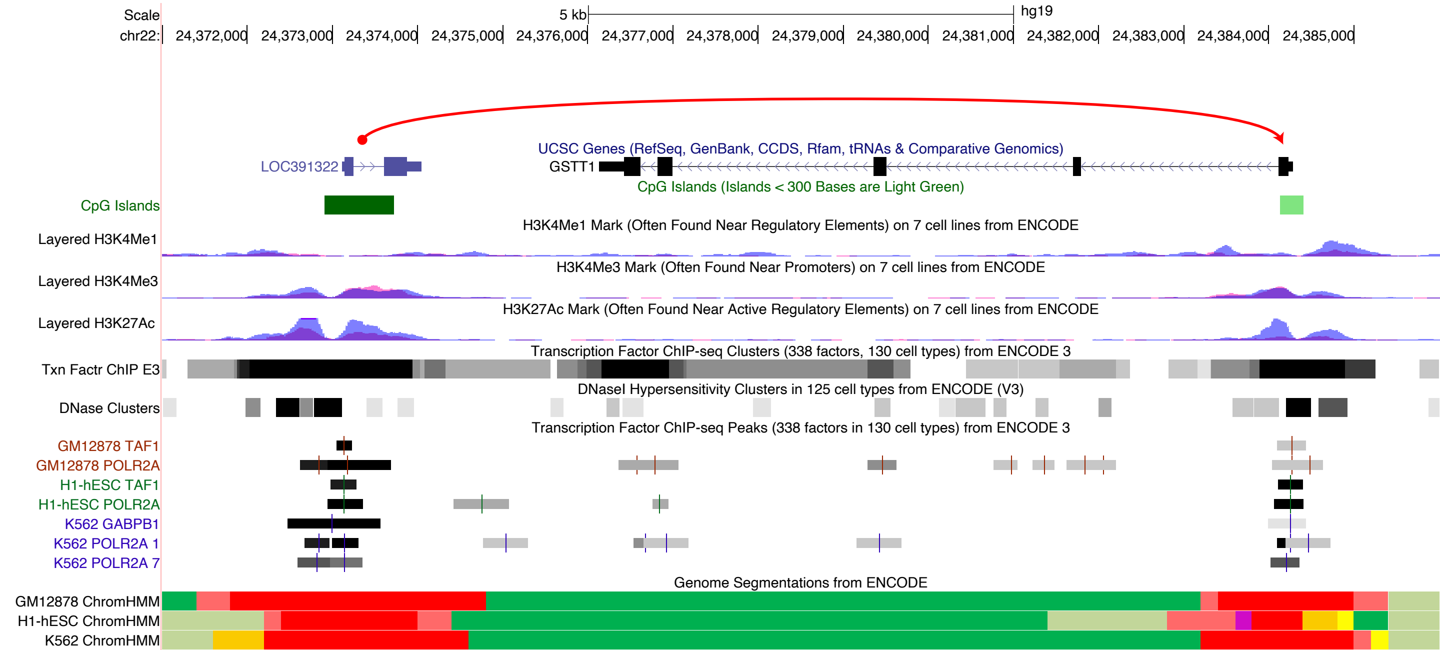

Figure S3: **An example of an activating MethNet association**. *GSTT1* is regulated by the promoter of a downstream non-coding gene. UCSC Genome Browser session highlighting the MethNet association on top track.

Supplementary Figure S4

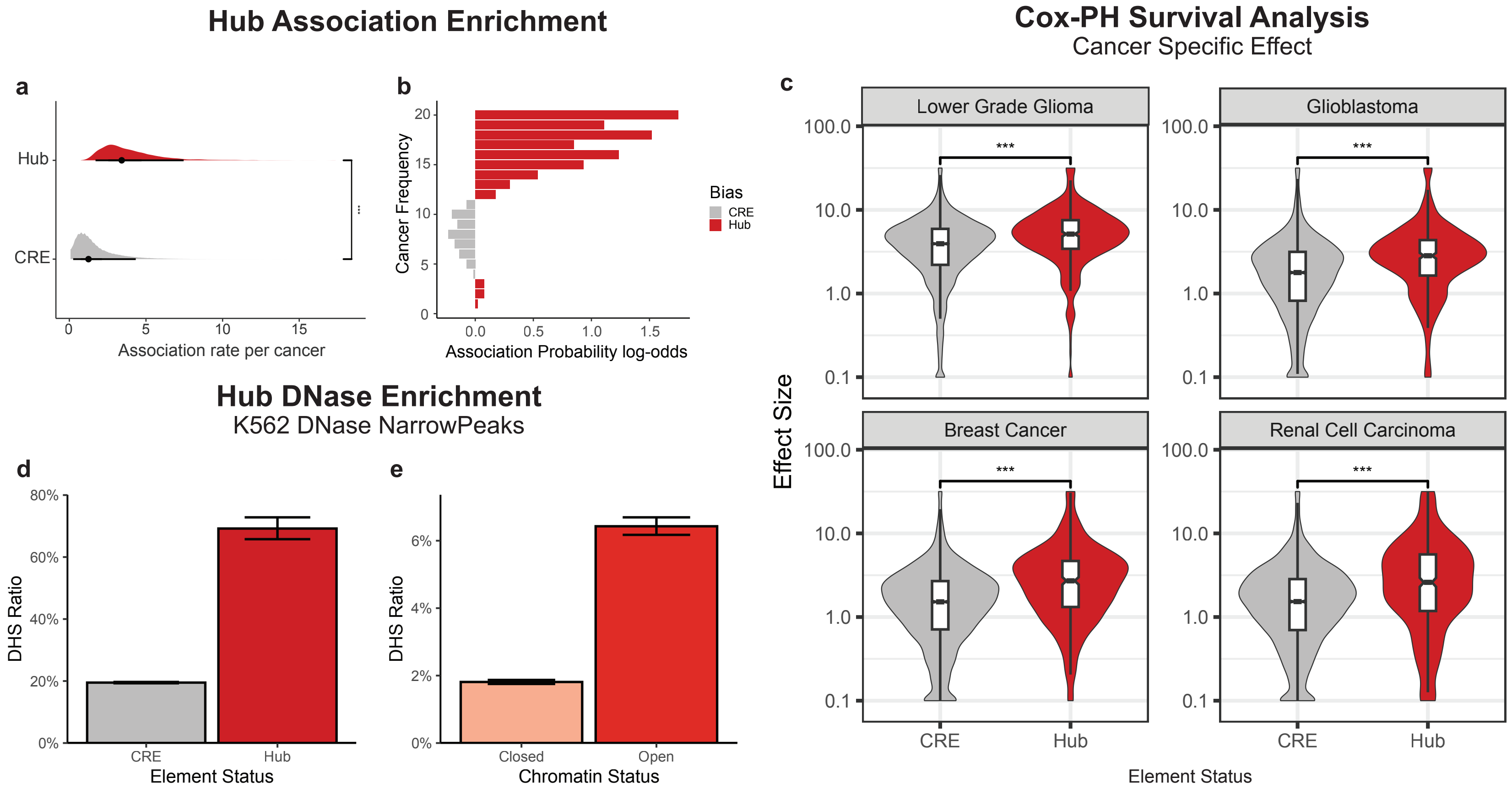

Figure S4. **Enrichment of Hubs elements relative to CREs**. **a**) Distributions of average associations per cancer for CREs that are characterized as hubs and the rest. A two-sided Wilcoxon rank sum test with continuity correction is used to compare the distributions (p-value < 2.22e-16). **b**) MethNet hubs are enriched in robust associations, that are recovered across multiple cancers, compared to regular CREs. Positive values along the x-axis represent that a higher ratio of hubs fall in the corresponding category compared to non-hub CREs. **c**) Cancer-specific effects of methylation of hubs (n=574) vs non-hub elements (n=174,027). Distribution of the absolute marginal effect methylation of hub and non-hubs elements for TCGA datasets that were affect more than the rest. Boxplots were drawn with the following parameters: box bounds correspond to 1st and 3rd quantiles, center mark corresponds to median, whiskers length is 1.5 the height of the box (inter-quantile region) or up to the extrema of the distribution if they are closer to the box bound. P-values were calculated using a two-sided Wilcoxon rank sum test with continuity correction ($p_{Lower\ Grade\ Glioma}$=6.4x10-5, $p_{Glioblastoma}$= $1.1x10^{-30}$, $p_{Breast\ Cancer}$=$5.8x10^{-24}$, $p_{Renal\ Cell\ Carcinoma}$=$6.6x10^{-8}$). **d**) Regulatory hubs are on average more likely to overlap open chromatin regions relative to non-hub elements. Bar height represents probability that a CRE/Hub overlaps a Dnase peak and error bars represent the 95% confidence interval (n_{Hub}=6139, n_{CRE}=239416). **e**) Elements overlapping open chromatin region are more likely to be hubs. Bar height represents probability an element overlapping a Closed/Open chromatin region is a Hub and error bars represent the 95% confidence interval (n_{Open}= 41576, n_{Closed}=203979).

Supplementary Figure S5

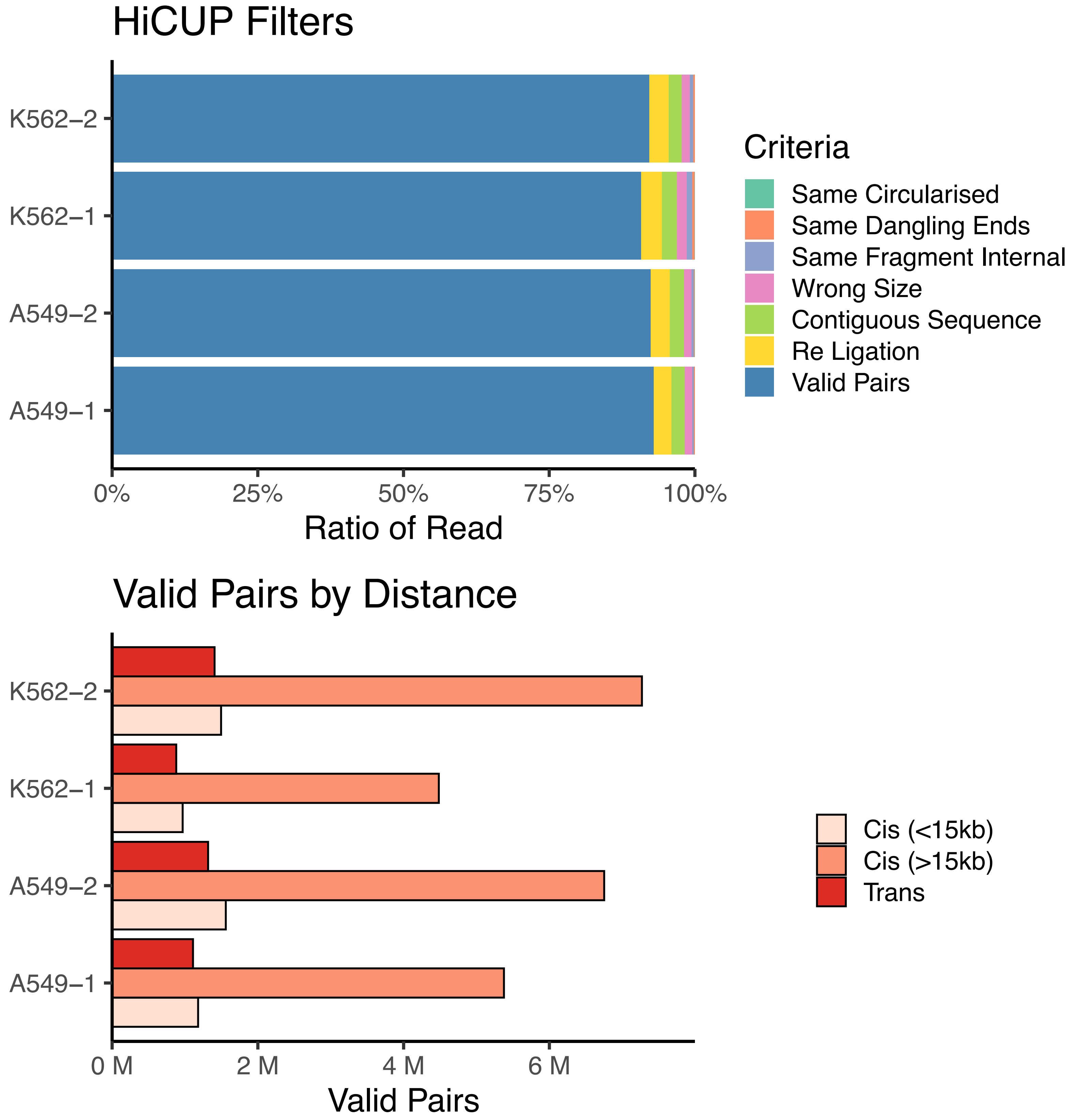

Figure S5: **Quality control metrics of promoter capture Hi-C reads**. Top: proportions of reads filtered out by HiCUP filtering steps. "Valid Pairs" were used for subsequent analysis. Bottom: Proportion of cis and trans valid pair reads. Cis pairs are categorized into short (less than 15kbp) and long (more than 15kbp) range valid pair reads.

Supplementary Figure S6

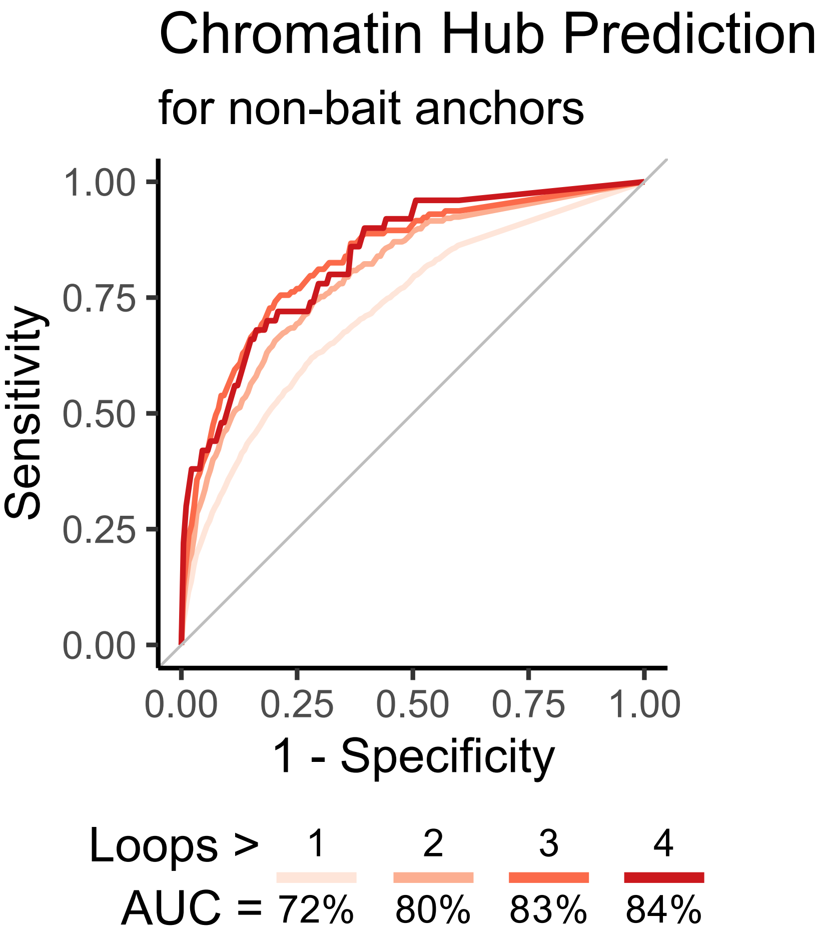

Figure S6: **MethNet potential predictive power for chromatin hubs**. We analyzed intergenic hubs separately from promoter hubs because the latter are enriched in our data because of the experimental design. As a result, we used thresholds that were more lenient.

Supplementary Figure S7

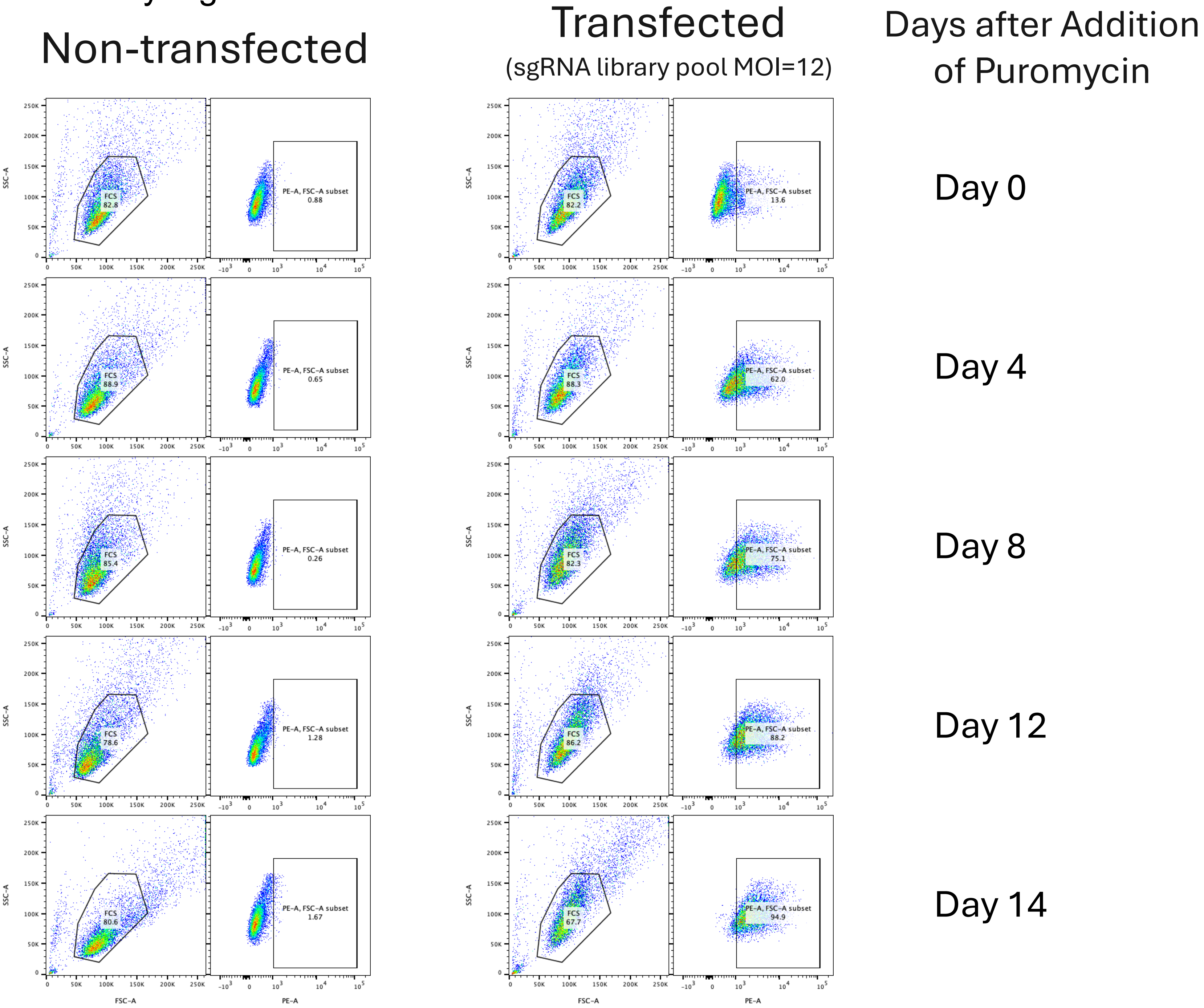

Figure S7**: RFP flow cytometry to assess the enrichment of cells transfected with A549-dCas9-KRAB-MeCP after puromycin selection for the perturb-seq assay**. Gates in the left panels correspond to the living cells while gates in the right panels to RFP (PE-A) positive cell population. Data are shown for day 0 to 14 after puromycine selection. At day 14, 95% of the cells were RFP positive cells in the sample transfected with the sgRNA library pool (MOI = 12), compared to 2% in the non-transfected control sample.

Supplementary Figure S8

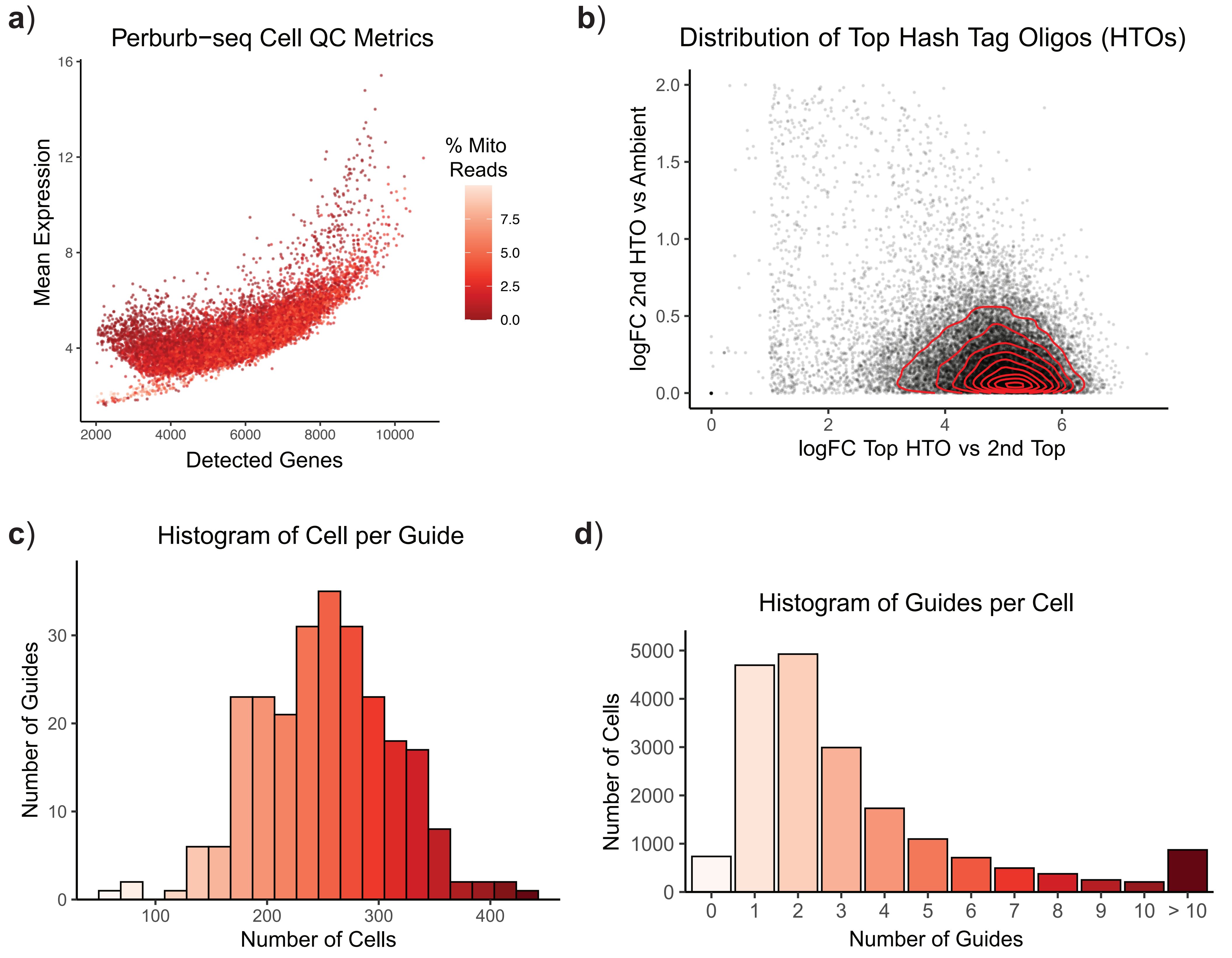

Figure S8: **Distribution of metrics used to select MethNet CRE to validate with perturb-seq**. **a**) Scatter plot of quality control metrics of the filtered cells. Points represent cells (n=19091), the horizontal axis represents number of genes detected in each cell, the vertical axis represents the aver expression (reads) per gene. **b**) Distribution of log2 fold change of the two most abundant oligo-tagged antibodies detected in each cell. **c**) Distribution of the number of cells per guide. **d**) Distribution of the number of guides per cell.

Supplementary Figure S9

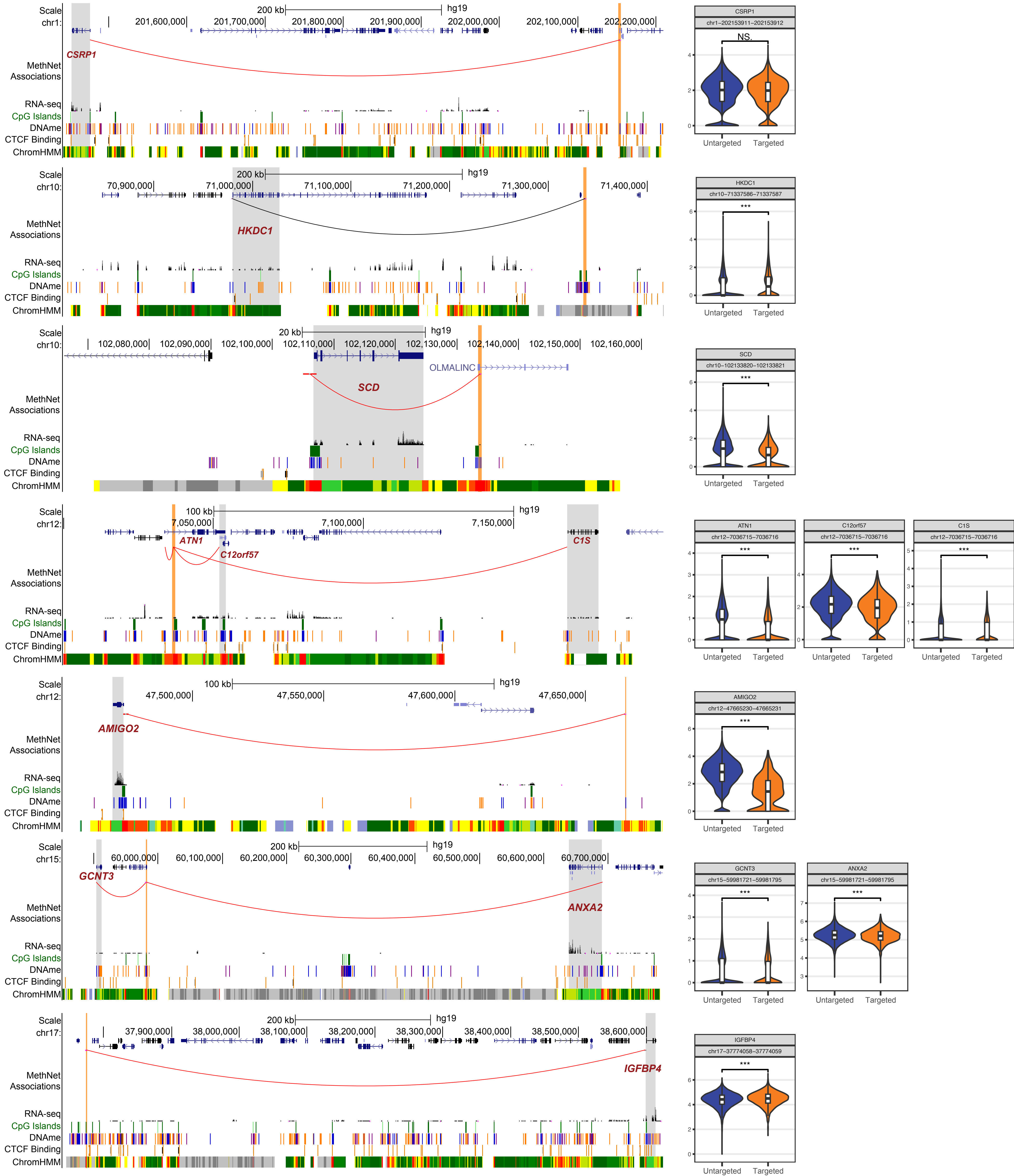

Figure S9. **All the association validated by perturb-seq**. Each panel shows the impact of targeting a regulatory region to the corresponding genes. Cells are grouped based on the detection of any sgRNA guide targeting the region. Boxplots were drawn with the following parameters: box bounds correspond to 1st and 3rd quantiles, center mark corresponds to median, whiskers length is 1.5 the height of the box (inter-quantile region) or up to the extrema of the distribution if they are closer to the box bound. P-values were calculated using a two-sided Welch two sample t-test. Precise p-values and sample sizes are given in Source Data.

Supplementary Figure S10

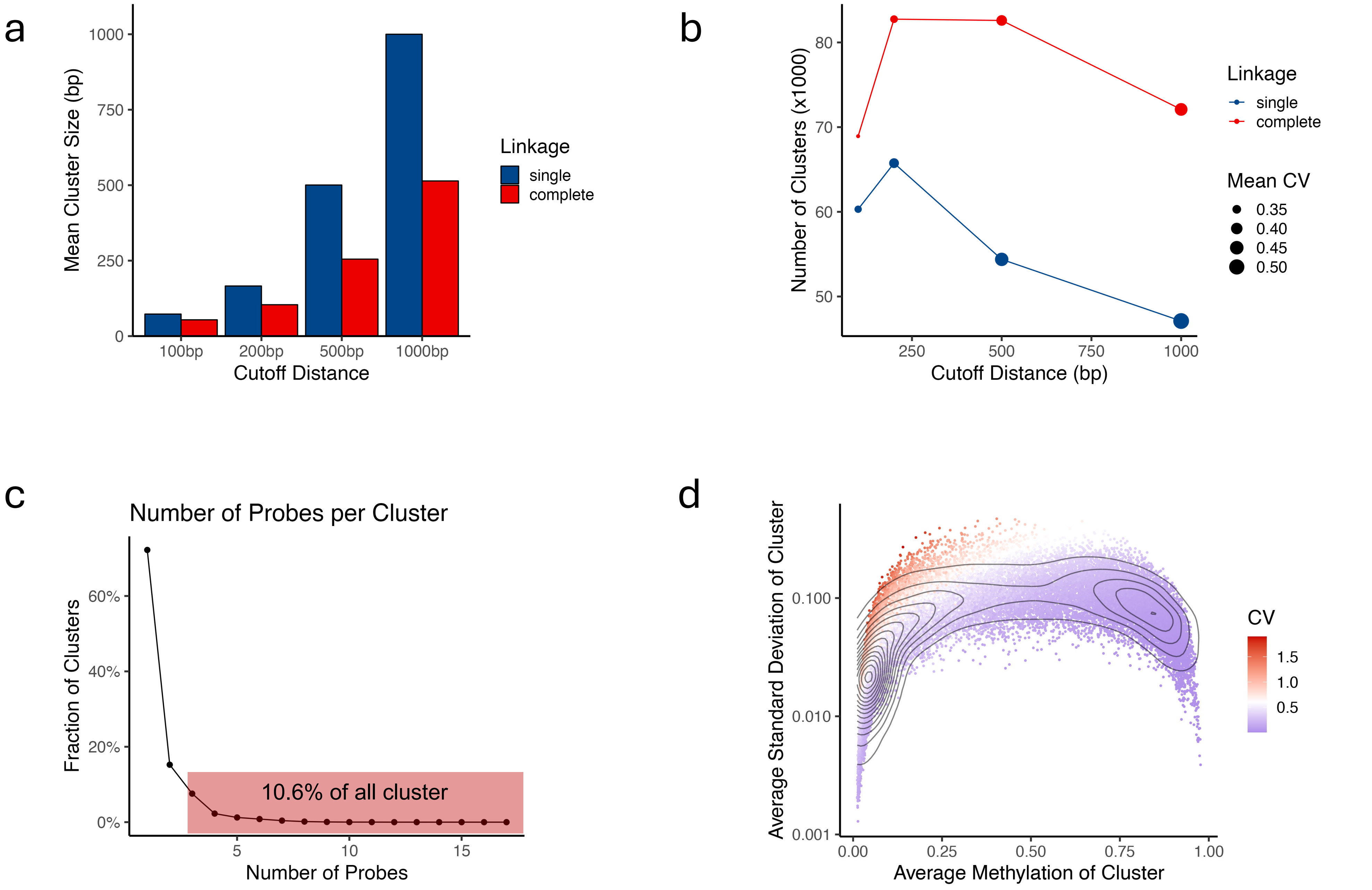

Figure S10: **CpG cluster selection and characterization**. a-b) Criteria for selecting linkage method and distance cutoff. c-d) Characterization of clusters used for MethNet (complete linkage with 200bp distance cutoff). a) Average size of CpG clusters for different cutoffs and linkages (ignoring singletons). b) Number of clusters and average coefficient of variation (CV) for different cutoffs and linkage methods. c) Distribution of Cluster Sizes. We used clusters with more than 2 CpG probes (shaded area) to calculate mean and standard deviation. d) Joint distribution of average standard deviation and fractional methylation of the clusters across all samples.

Supplementary Figure S11

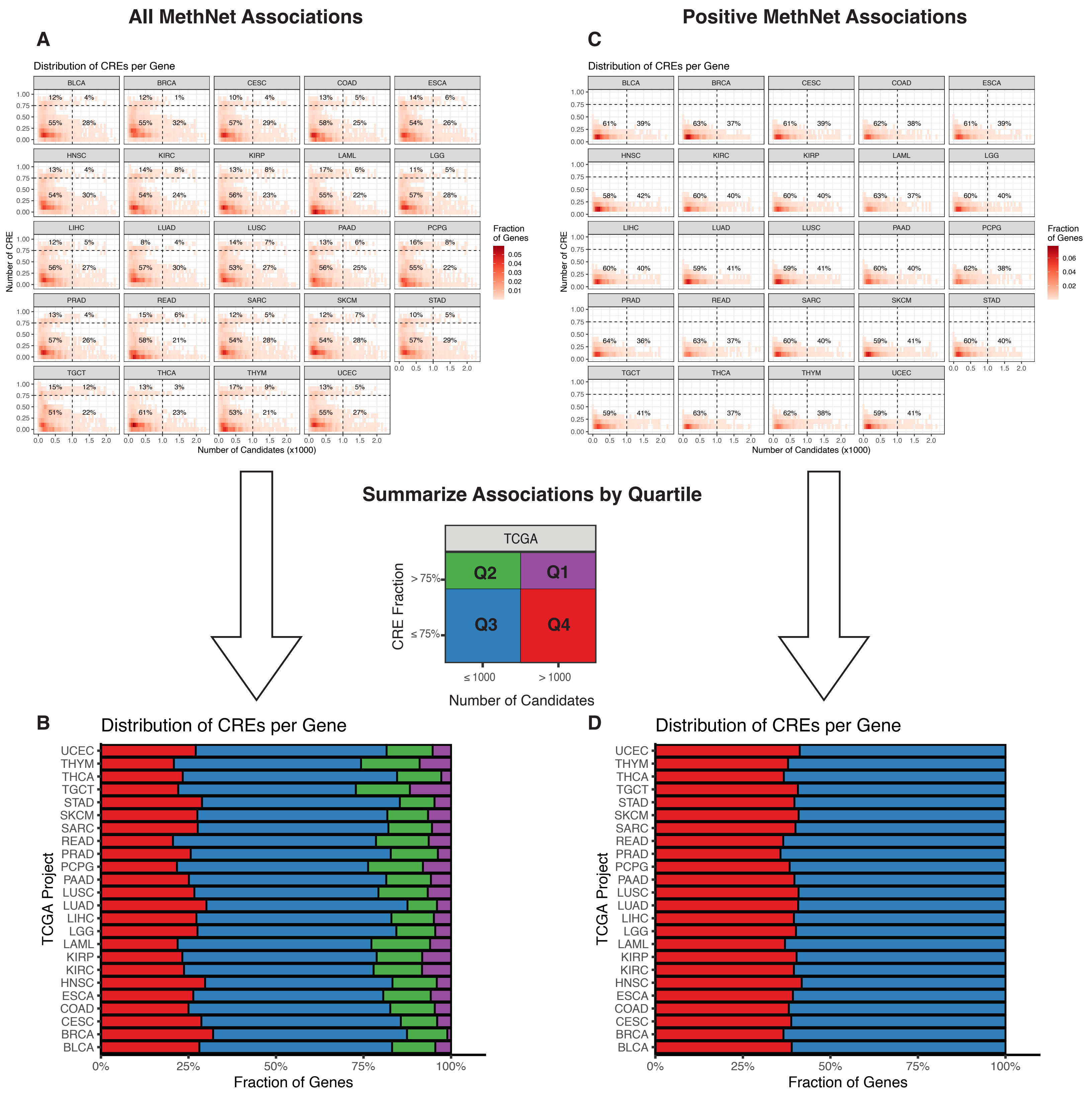

Figure S11: **Joined distribution of the number of CRE candidates per gene and the fraction of CREs selected by MethNet**. The left panels are the distributions of all associations recovered by MethNet, while the right panels reflect the distributions of the associations with a positive MethNet score (which defines “MethNet CREs”). The bottom panels summarize the information in the top panels by quantile as shown by the common legend in the middle. MethNet associations in which most (>75%) of the candidates were identified as CREs correspond to Q2 and Q1 which were not present (or negligible) among high-confidence CREs.
