# Supplementary material for: MethNet: a robust approach to identify regulatory hubs and their distal targets from cancer data
Source: Nat Commun. 2024 Jul 17;15:6027. doi: 10.1038/s41467-024-50380-3 (PMC11258126; doi:10.1038/s41467-024-50380-3)
Supplement: Supplementary file 3 — Description of Additional Supplementary Files [file 41467_2024_50380_MOESM3_ESM.pdf]

<u>Description of Additional Supplementary Files</u>

Supplementary Data 1

methnet.csv
Combines the results from the initial step into the consensus regulatory network. Columns:
gene: name of gene regulated
cluster: name and coordinates of CRE
dist: average distance between CpG probes in CRE and gene TSS
overlap: whether the CRE overlaps the gene body
coef: mean coefficient of the association across all TCGA studies
nup: number of TCGA studies where the association was silencer-like (methylation leads to up-regulation)
ndn: number of TCGA studies where the association was enhancer-like (methylation leads to down-regulation)
ncancer: number of TCGA studies where the association was detectable.
contrib: relative contribution of association to gene regulation
score: MethNet score of associations
promoter: whether cluster is in the promoter of the gene
intergenic: whether the association is intergenic (and used to compute cluster_score.csv)

cluster_score.csv
The results of the analysis of the intergenic CRE. Columns:
cluster: name and coordinates of CRE
Hub: whether the CRE is a Hub or not
score: MethNet regulatory potential
ninter: number of interactions (degree of node)
nup: number of silencer-like interactions (methylation up-regulates)
ndn: number of enhancer-like interactions (methylation down-regulates)
score_rank: relative ranking of score (1 = highest score)

Perturb-seq design.xlsx

Data used to select and design the sgRNA to target potential CREs. See first spreadsheet (README) for information about columns.
