# Supplementary material for: MethNet: a robust approach to identify regulatory hubs and their distal targets from cancer data
Source: Nat Commun. 2024 Jul 17;15:6027. doi: 10.1038/s41467-024-50380-3 (PMC11258126; doi:10.1038/s41467-024-50380-3)
Supplement: Supplementary file 5 — Reporting Summary [file 41467_2024_50380_MOESM5_ESM.pdf]

nature portfolio

Corresponding author(s): Jane A Skok, Aristotelis Tsirigos

Last updated by author(s): Jun 21, 2024

Reporting Summary

Nature Portfolio wishes to improve the reproducibility of the work that we publish. This form provides structure for consistency and transparency in reporting. For further information on Nature Portfolio policies, see our Editorial Policies and the Editorial Policy Checklist.

Statistics

For all statistical analyses, confirm that the following items are present in the figure legend, table legend, main text, or Methods section.

n/a	Confirmed	
☐	☒	The exact sample size (*n*) for each experimental group/condition, given as a discrete number and unit of measurement
☐	☒	A statement on whether measurements were taken from distinct samples or whether the same sample was measured repeatedly
☐	☒	The statistical test(s) used AND whether they are one- or two-sided<br>*Only common tests should be described solely by name; describe more complex techniques in the Methods section.*
☐	☒	A description of all covariates tested
☐	☒	A description of any assumptions or corrections, such as tests of normality and adjustment for multiple comparisons
☐	☒	A full description of the statistical parameters including central tendency (e.g. means) or other basic estimates (e.g. regression coefficient) AND variation (e.g. standard deviation) or associated estimates of uncertainty (e.g. confidence intervals)
☐	☒	For null hypothesis testing, the test statistic (e.g. *F*, *t*, *r*) with confidence intervals, effect sizes, degrees of freedom and *P* value noted<br>*Give P values as exact values whenever suitable.*
☒	☐	For Bayesian analysis, information on the choice of priors and Markov chain Monte Carlo settings
☒	☐	For hierarchical and complex designs, identification of the appropriate level for tests and full reporting of outcomes
☐	☒	Estimates of effect sizes (e.g. Cohen's *d*, Pearson's *r*), indicating how they were calculated

Our web collection on statistics for biologists contains articles on many of the points above.

Software and code

Policy information about availability of computer code

Data collection	Data collection is automated and the code to reproduce it together with version numbers is provided in the GitHub page that accompanies this study: https://github.com/TeoSakel/MethNet. Zenodo DOI: 10.5281/zenodo.11404065
Data analysis	The code used to analyze the data is available at https://github.com/TeoSakel/MethNet. Zenodo DOI: 10.5281/zenodo.11404065

For manuscripts utilizing custom algorithms or software that are central to the research but not yet described in published literature, software must be made available to editors and reviewers. We strongly encourage code deposition in a community repository (e.g. GitHub). See the Nature Portfolio guidelines for submitting code & software for further information.

Data

Policy information about availability of data

All manuscripts must include a data availability statement. This statement should provide the following information, where applicable:

- Accession codes, unique identifiers, or web links for publicly available datasets
- A description of any restrictions on data availability
- For clinical datasets or third party data, please ensure that the statement adheres to our policy

The results published here are in part based upon data generated by the TCGA Research Network [https://www.cancer.gov/tcga]. We collected paired gene expression and methylation data via the xenahubs portal69 which downloaded data from gdc.cancer.gov (data release 9.0 - October 24, 2017). We used the pan-cancer batch-corrected normalized gene expression [https://www.synapse.org/#!Synapse:syn4976369] and beta values for methylation from Illumina's

HumanMethylation450 BeadChip [https://www.synapse.org/#!Synapse:syn4557906]. Clinical data for those samples was downloaded from xenahubs where available [https://www.synapse.org/#!Synapse:syn8402823]. Metadata for the CpG probes were collected from Illumina's annotation of HumanMethhylation450 BeadChip via the IlluminaHumanMethylation450kanno.ilmn12.hg19 Bioconductor package.
The annotation was augmented (using the custom script annotate_clusters.R) by overlapping clusters with tracks from the UCSC genome browser. We used the ChromHMM chromatin annotation, CG island, and the transcription factor binding site cluster tracks. Links for all the data downloaded for this annotation are included in the custom script. Annotations were based on the most common labeling across all cell types.
DNAse data for K562 cells were downloaded from ENCODE, we used the DNase regions of the combined replicates (file id ENCFF621ZJY).
Hi-ChIP loops were downloaded from the supplementary material of the FitHiChIP paper34. We used the combined loose and merged replicate (L+M) loops for 2.5kb bins for all cell lines (CD4-Naive, GM12878, K562). The primary data for Hi-ChIP loops were generated by Mumbach MR et al.,71 [https://www.ncbi.nlm.nih.gov/geo/query/acc.cgi?acc=GSE101498]. The manually processed data are shared at the GitHub repository of MethNet (see Code Availability) as Bhattacharyya_loops.csv.gz.
The raw and processed sequencing data generated in this study have been submitted to the Gene Expression Omnibus (GEO) database under the superfamily accession number GSE236305 [https://www.ncbi.nlm.nih.gov/geo/query/acc.cgi?acc=GSE236305]. The promoter capture Hi-C and Perturb-seq data accession numbers are GSE235851 [https://www.ncbi.nlm.nih.gov/geo/query/acc.cgi?acc=GSE235851] and GSE236304 [https://www.ncbi.nlm.nih.gov/geo/query/acc.cgi?acc=GSE236304], respectively. The results of MethNet analysis used to generate the figures are uploaded to figshare [https://doi.org/10.6084/m9.figshare.25988074.v3].
No previously published data are under restricted access.
Source data are provided with this paper.

Research involving human participants, their data, or biological material

Policy information about studies with human participants or human data. See also policy information about sex, gender (identity/presentation), and sexual orientation and race, ethnicity and racism.

Reporting on sex and gender	All preprocessing and normalization of DNA methylation and RNA-seq data was performed by TCGA/Xenahubs consortiums. Our analysis is sex/gender agnostic, sex chromosome were removed from downstream analysis which is common practice in DNA methylation analysis since inbalance of Y-chromosomes and X-inactivation lead to confounding effects
Reporting on race, ethnicity, or other socially relevant groupings	No socially constructed or relevant variables were used for this analysis
Population characteristics	Population characteristic were are included in the clinical data downloaded for this analysis which are based on: https://www.synapse.org/#!Synapse:syn8402823
Recruitment	No primary human samples were collected for this study. We used all the samples that had matching DNA methylation and RNA-seq data.
Ethics oversight	No organization approved the study protocol. The study was based on publicly available data.

Note that full information on the approval of the study protocol must also be provided in the manuscript.

Field-specific reporting

Please select the one below that is the best fit for your research. If you are not sure, read the appropriate sections before making your selection.

☒ Life sciences ☐ Behavioural & social sciences ☐ Ecological, evolutionary & environmental sciences

For a reference copy of the document with all sections, see nature.com/documents/nr-reporting-summary-flat.pdf

Life sciences study design

All studies must disclose on these points even when the disclosure is negative.

Sample size	No sample size calculation were performed as we did not aim to identify a specific effect size. Sample sizes were determined based on the assay provider suggestions (Arima and Cellecta) with the aim to explore the loop/perturbation landscape of the cells.
Data exclusions	No data were excluded from the analysis.
Replication	We followed the assay provider protocols to guarantee experimental reproducibility. Those included 2 technical replicates per cell line in the case of promoter capture HiC and multiple guides per target in the case of promoter capture HiC.
Randomization	Patients samples were split into tumor and normal/metastatic samples based on TCGA annotations when performing the analysis. MethNet associations were ranked and hubs were called based on a percentile threshold.
Blinding	Blinding is not relevant for this study since it did not include human subjects and researcher need to know the labels in order to perform the analysis.

Reporting for specific materials, systems and methods

We require information from authors about some types of materials, experimental systems and methods used in many studies. Here, indicate whether each material, system or method listed is relevant to your study. If you are not sure if a list item applies to your research, read the appropriate section before selecting a response.

Materials & experimental systems

n/a	Involved in the study
☒	☐ Antibodies
☐	☒ Eukaryotic cell lines
☒	☐ Palaeontology and archaeology
☒	☐ Animals and other organisms
☒	☐ Clinical data
☒	☐ Dual use research of concern
☒	☐ Plants

Methods

n/a	Involved in the study
☒	☐ ChIP-seq
☐	☒ Flow cytometry
☒	☐ MRI-based neuroimaging

Eukaryotic cell lines

Policy information about cell lines and Sex and Gender in Research

Cell line source(s)	The A549 and K562 cell lines were purchased from ATCC catalog number: CCL-185 and CRL-3343, respectively. K562 cell line is derived from a female patient with erythromyeloid leukemia, A549 from a male patient with lung cancer.
Authentication	None of the cell lines were authenticated
Mycoplasma contamination	All cell lines were tested for mycoplasma using the Universal Mycoplasma Detection Kit (30-1012K, ATCC) and they were all negative.
Commonly misidentified lines (See ICLAC register)	No commonly misidentified lines were used in this study

Plants

Seed stocks	*Report on the source of all seed stocks or other plant material used. If applicable, state the seed stock centre and catalogue number. If plant specimens were collected from the field, describe the collection location, date and sampling procedures.*
Novel plant genotypes	*Describe the methods by which all novel plant genotypes were produced. This includes those generated by transgenic approaches, gene editing, chemical/radiation-based mutagenesis and hybridization. For transgenic lines, describe the transformation method, the number of independent lines analyzed and the generation upon which experiments were performed. For gene-edited lines, describe the editor used, the endogenous sequence targeted for editing, the targeting guide RNA sequence (if applicable) and how the editor was applied.*
Authentication	*Describe any authentication procedures for each seed stock used or novel genotype generated. Describe any experiments used to assess the effect of a mutation and, where applicable, how potential secondary effects (e.g. second site T-DNA insertions, mosiacism, off-target gene editing) were examined.*

Flow Cytometry

Plots

Confirm that:

☒ The axis labels state the marker and fluorochrome used (e.g. CD4-FITC).

☒ The axis scales are clearly visible. Include numbers along axes only for bottom left plot of group (a 'group' is an analysis of identical markers).

☒ All plots are contour plots with outliers or pseudocolor plots.

☒ A numerical value for number of cells or percentage (with statistics) is provided.

Methodology

Sample preparation

A549 cells were transduced with lentiviruses expressing dCas9-KRAB-MECP plasmids as described in the Methods section. The cells were then grown in DMEM medium (Gibco/Invitrogen) + 10% FBS + 100 units/ml penicillin + 100 µg/ml streptomycin + 5% CO2 at 37. After 1 week of culture, the cells were tested for mycoplasma using the Universal Mycoplasma Detection Kit (30-1012K, ATCC)

On the day 0, the cells were transduced with the sgRNA lentiviral library for Perturb-seq at the concentration of 10ug/ml of LentiTrans Reagent. The medium was changed after after 24 hours post-transduction without addition of LentiTrans Reagent. On Day 3, 1ug/ml puromycin were added to select cells successfully transduced with the sgRNA. On the day of collection (day 14), the cells were trypsinized and a 300 ul aliquot of cells was suspended in PBS buffer containing 2% FBS for

flow cytometry to assess the percentage of transduced cells (more than 90%).

Instrument	BD LSR II Flow Cytometer
Software	FlowJo
Cell population abundance	Flow cytometry was performed as quality control to assess the abundance of the relevant cells without sorting prior to the perturb-seq experiment. The relevant cell population was more than 90% (RFP/PE positive cells) as quantified by Flow cytometry.
Gating strategy	Preliminary FSC/SCC gating was performed using FlowJo to exclude cell debris based on their low FSC and SCC values (lower left corner). Non-tranfected cells were used as negative control for RFP/PE signal, the positive gating was performed so that less than 2 % of the non-transfected cells were positive for RFP/PE.

☒ Tick this box to confirm that a figure exemplifying the gating strategy is provided in the Supplementary Information.
